# Vindoline Inhibits RANKL-Induced Osteoclastogenesis and Prevents Ovariectomy-Induced Bone Loss in Mice

**DOI:** 10.3389/fphar.2019.01587

**Published:** 2020-01-22

**Authors:** Yunfei Zhan, Jiamin Liang, Kun Tian, Zhigang Che, Ziyi Wang, Xue Yang, Yuangang Su, Xixi Lin, Fangming Song, Jinmin Zhao, Jiake Xu, Qian Liu, Bo Zhou

**Affiliations:** ^1^ Guangxi Key Laboratory of Regenerative Medicine, Guangxi Medical University, Nanning, China; ^2^ School of Biomedical Sciences, The University of Western Australia, Perth, WA, Australia; ^3^ Department of Trauma Orthopedic and Hand Surgery, Research Centre for Regenerative Medicine, The First Affiliated Hospital of Guangxi Medical University, Nanning, China

**Keywords:** vindoline, MAPK, osteoclast, osteoporosis, NFATc1

## Abstract

Osteolytic bone diseases, for example postmenopausal osteoporosis, arise from the imbalances between osteoclasts and osteoblasts in the bone remodeling process, whereby osteoclastic bone resorption greatly exceeds osteoblastic bone formation resulting in severe bone loss and deterioration in bone structure and microarchitecture. Therefore, the identification of agents that can inhibit osteoclast formation and/or function for the treatment of osteolytic bone disease has been the focus of bone and orthopedic research. Vindoline (Vin), an indole alkaloid extracted from the medicinal plant *Catharanthus roseus*, has been shown to possess extensive biological and pharmacological benefits, but its effects on bone metabolism remains to be documented. Our study demonstrated for the first time, that Vin could inhibit osteoclast differentiation from bone marrow macrophages (BMMs) precursor cells as well as mature osteoclastic bone resorption. We further determined that the underlying molecular mechanism of action of Vin is in part due to its inhibitory effect against the activation of MAPK including p38, JNK, and ERK and intracellular reactive oxygen species (ROS) production. This effect ultimately suppressed the induction of c-Fos and NFATc1, which consequently downregulated the expression of the genes required for osteoclast formation and bone resorption. Consistent with our *in vitro* findings, *in vivo* administration of Vin protected mice against ovariectomy (OVX)-induced bone loss and trabecular bone deterioration. These results provided promising evidence for the potential therapeutic application of Vin as a novel treatment option against osteolytic diseases.

## Introduction

Bone is a dynamic tissue that constantly undergoes remodeling *via* osteoblastic bone formation and osteoclastic bone resorption (Saito et al., 2019). This balanced homeostatic process ensures the continuous renewal and repair of bone tissue keeping it in an optimal working condition. Inevitably, various circumstances including environmental, physical, chemical, or metabolic changes can shift this balance towards elevated bone resorption, leading to excessive bone loss and deterioration of bone architecture (Del Puente et al., 2015). Compromised bone stability and bone strength often leads to increased fracture risks that seriously affect the quality of life and increase mortality rate of suffering patients (Xi et al., 2019). Postmenopausal osteoporosis is the most common form of osteoporotic bone loss, where one-third of the women over the age of 50 and more than 200 million people worldwide suffered from the disease (Reginster and Burlet, 2006; Harvey et al., 2010; Rachner et al., 2011).

Osteoclast differentiation from monocytic precursors of the hematopoietic stem cell lineage is a sequential process under the control of two key cytokines, macrophage colony-stimulating factor (M-CSF) and receptor activator of nuclear factor-κB ligand (RANKL) (Teitelbaum, 2007). Binding of RANKL to receptor RANK on monocytic precursors initiates the activation of downstream signaling pathways and second messengers systems of which MAPK and nuclear factor-κB (NF-κB) pathways are most prominent (Asagiri and Takayanagi, 2007). These pathways synergistically induce the expression and activation of transcription factors, c-Fos and NFATc1, the latter is the definitive factor governing osteoclast differentiation (Takayanagi et al., 2002). Many osteoclast marker genes including those involved in osteoclast fusion and bone resorption are under the transcriptional control of NFATc1 (Sundaram et al., 2007). Hence, the RANKL-RANK signaling axis has been the prime target for identification or development of inhibitory agents for the therapeutic application in osteolytic conditions such as osteoporosis.

There have been growing interests in the search for naturally derived chemical agents and compounds that possess anti-osteoclastogenic and/or anti-resorptive properties in recent years. Vin is an indole alkaloid extracted from the medicinal plant *Catharanthus roseus* which has been shown to have anti-tumor, anti-diabetic, anti-apoptotic, anti-oxidant, and anti-inflammatory effects (Rasineni et al., 2010; Goboza et al., 2019). In spite of its impressive repertoire of biological benefits, the effect of Vin on osteoclast remains to be determined. Our present study found that Vin attenuated BMM-derived osteoclast formation as well as mature osteoclast bone resorptive function *in vitro*. Mechanistically we determined that Vin suppressed the activation of all three MAPK signaling pathways and intracellular ROS production consequently resulting in the repression of c-Fos and NFATc1 expression. Using a well-established model of ovariectomy (OVX)-induced bone loss in mice, we provided further evidence that Vin administration protected mice from the harmful effect of OVX on bone. In conclusion, these results provided evidence for the potential therapeutic application of Vin in osteolytic bone diseases such as osteoporosis.

## Materials and Methods

### Chemicals, Media, and Reagents

Compound Vin with a purity >98% was obtained from Chengdu Must Bio‐Technology Co., Ltd (Chengdu, Sichuan Province, China). α‐modified minimal essential medium (α‐MEM), penicillin–streptomycin solution and fetal bovine serum were purchased from Gibco (Thermo Fisher Scientific, Waltham, MA, USA). Cell Counting Kit‐8 (CCK-8) was bought from Sigma‐Aldrich (St Louis, MO, USA). The high affinity actin probe Rhodamine-conjugated phalloidin was from Molecular Probes (Eugene, OR, USA). RANKL and M-CSF were bought from PeproTech (Princeton, NJ, USA). All primary antibodies and second antibodies were obtained from Cell Signaling Technology (Danvers, MA, USA). Reactive Oxygen Species (ROS) Assay Kit was acquired from Beyotime Institute of Biotechnology (Jiangsu, China).

### Osteoclast Culture and Differentiation Assay

Bone marrow macrophages (BMMs) were obtained by marrow flushing of femur and tibia excised from 6-week-old C57BL/6 mice. BMMs were cultured in α-MEM supplemented with 10% FBS, 1% penicillin-streptomycin solution, and 50 ng/ml M-CSF (referred to as complete α-MEM). Media were replenished every 2 days and the adherent BMMs were considered as osteoclast precursor cells that were used for subsequent experiments. For osteoclast differentiation, BMMs were seeded in the medium at a density of 6×10^3^ cells/well and were allowed to adhere to the well surface overnight. The cells were then stimulated with 100 ng/ml RANKL without (mock control) or with various concentrations of Vin (1.25, 2.5, 5, or 10 μM). Media containing RANKL and Vin were replenished every 2 days for 5 days. Then the cells were stained for tartrate-resistant acid phosphatase (TRAP) activity assay. Micrographs of TRAP-stained cells were acquired using a light microscope, and the number and area of osteoclasts were quantified using ImageJ software (NIH, Bethesda, MD, USA).

### Cytotoxicity Assay

The cytotoxic effect of Vin on BMM cell viability was examined using the CCK-8 Assay Kit. Briefly, BMMs seeded in 96-well plates in triplicates were treated without (mock control) or with different concentrations of Vin (1.25, 2.5, 5, 10, 20, or 40 μM) for 48 h. The cells were then incubated with CCK-8 reagent for 2 h, and the absorbance at the wavelength of 450 nm was measured using a Multiskan Spectrum microplate spectrophotometer (Thermo Fisher Scientific).

### Intracellular ROS Generation Assay

Intracellular ROS production following RANKL stimulation was determined using DCFDA cellular ROS Assay Kit as per manufacturer's instruction and as previously described with minor modifications (Bae et al., 1997; Lee et al., 2005). In brief, BMMs were stimulated with RANKL without or with 5 or 10 μM of Vin for 48 h. Cells were washed quickly and then incubated with dichloro-dihydro-fluorescein diacetate (DCFH-DA) diluted 1:1,000 in HANKS buffer for 40 min. The oxidative conversion of DCFH-DA to highly fluorescent DCF by ROS was detected under fluorescence microscope. The mean fluorescence intensity was analyzed using ImageJ software (NIH).

### Podosomal F-Actin Belt Immunofluorescence

To determine whether Vin affects the actin cytoskeleton, we performed F-actin staining on BMM-derived osteoclasts stimulated with RANKL without (mock control) or with 5 or 10 μM of Vin treatment for 5 days. After fixation with 4% paraformaldehyde (PFA) and permeabilization with 0.1% Triton X-100, cells were then stained with Rhodamine-conjugated phalloidin for 1 h. Nuclei were counterstained with DAPI for 5 min. Fluorescence images were acquired on a fluorescence microscope.

### *In Vitro* Hydroxyapatite Resorption Assay

M-CSF-dependent BMMs were induced to form pre-osteoclasts by stimulation with RANKL for 3 days. The cells were then collected and reseeded in hydroxyapatite coated OsteoAssay plates (Corning Inc, Corning, NY, USA). Cells were allowed to settle and adhere to wells before treatment without (mock control) or with 5 or 10 μM of Vin. The treatment maintained for 48 h, with changing media once during the treatment. Finally, after the cells were removed, the resorption pits were observed under optical microscope and the percentage of the resorption area in the total area was quantified by ImageJ software.

### Quantitative Real-Time Polymerase Chain Reaction (qPCR)

BMMs were cultured in 6-well plates and stimulated with RANKL with or without the addition of 5 or 10 μM of Vin for 5 days until the mature osteoclasts were observed in the Vin-free control group. Total RNA was extracted using TRIzol reagent (Life Technologies, Carlsbad, CA, USA). RevertAid First Strand cDNA Synthesis Kit (Thermo Fisher Scientific) was used to reversely transcribe to complementary DNA (cDNA) from 1 μg of extracted total RNA in accordance with manufacturer's protocol (Thermo Fisher Scientific, Scoresby, Australia). cDNA was then used as template for qPCR using SYBR Green PCR Master Mix. Specific primers sequences were shown in **Table S1 (Supplementary Material)**. The expression of these genes was normalized to the expression of internal housekeeping gene GAPDH using 2^−ΔΔCT^ method.

### Protein Extraction and Western Blot Assay

To examine the effects of Vin on early events of RANKL-activated signaling pathways, BMMs were serum-free starved for 3 h, pretreated without or with 10 μM Vin for 1 h, and then stimulated with 100 ng/ml RANKL for 5, 10, 20, 30 or 60 min. For late RANKL-activated signaling events, BMMs were stimulated with RANKL in the absence or presence of 10 μM Vin for 1, 3, or 5 days. The cells without RANKL and Vin treatment were used as untreated 0 time point control. The total cellular proteins (TCPs) were extracted and then separated by SDS-PAGE gel and transferred onto nitrocellulose membranes (Thermo Fisher Scientific, Shanghai, China). Membranes were blocked for non-specific immunoreactivity by incubation in 5% BSA for 1 h and then incubated with primary antibodies for 12 h with gentle shaking. After extensive washes, membranes were incubated with corresponding secondary antibodies for 1 h in the dark. Western blot images were acquired on an ImageQuant LAS-4000 System (GE Healthcare, Chicago, IL, USA).

### Murine Model of Ovariectomy

All experiments were approved by the Ethics Committee of Guangxi Medical University. All animal experiments and surgical procedures were performed according to the guidelines of the Animal Care Committee of Guangxi Medical University. Twenty-four 11-week-old C57BL/6J female mice were randomly divided into 4 groups (6 mice in each group): Sham-operated (with normal saline injection), bilateral OVX group (with normal saline injection), OVX + low-dose Vin group (5 mg/kg body weight), and OVX + high-dose Vin group (10 mg/kg body weight). All mice were anesthetized with a solution of chloral hydrate, and received sham or OVX operation where a 0.5–0.8 cm incision was made along the midline of the back to remove the ovaries and part of the fallopian tube. Seven days later post-operation, mice were intraperitoneally injected every other day with either normal saline (Sham and OVX groups) or with Vin (low- and high-dose groups) for 6 weeks. Finally, all mice were sacrificed and tibias were resected for micro computed tomography (micro-CT) and histological assessment.

### Micro-CT Analyses

Three-dimensional (3D) reconstructions of the tibial bone from each group were generated from images acquired on a high-resolution SCANCO μCT100 scanner (SCANCO Medical AG, Brüttisellen, Switzerland). A square region of interest (ROI) 0.5 mm below the growth plate were selected for analysis. The following morphometric parameters of bone volume to total volume (BV/TV), trabecular number (Tb. N), trabecular separation (Tb. Sp) and trabecular thickness (Tb. Th) were analyzed by SCANCO Evaluation software V6.5.3.

### Histology and Histomorphometry Assessments

Following micro-CT analyses, tibias were decalcified with 12% EDTA and were embedded in paraffin blocks, which were then cut into sequential 5 μm sections for staining to detect TRAP activity. Sections were imaged under light microscopy at 40× and 100× magnification.

### Statistical Analysis

In this study, all data obtained from three or more independent experiments were analyzed by Student' s t-test and presented as mean ± standard deviation (SD). The results with p-values < 0.05 were considered to be statistically significant (95% confidence interval).

## Results

### Vin Attenuated Osteoclast Differentiation and Intracellular ROS Production *In Vitro*

**Figure 1:**
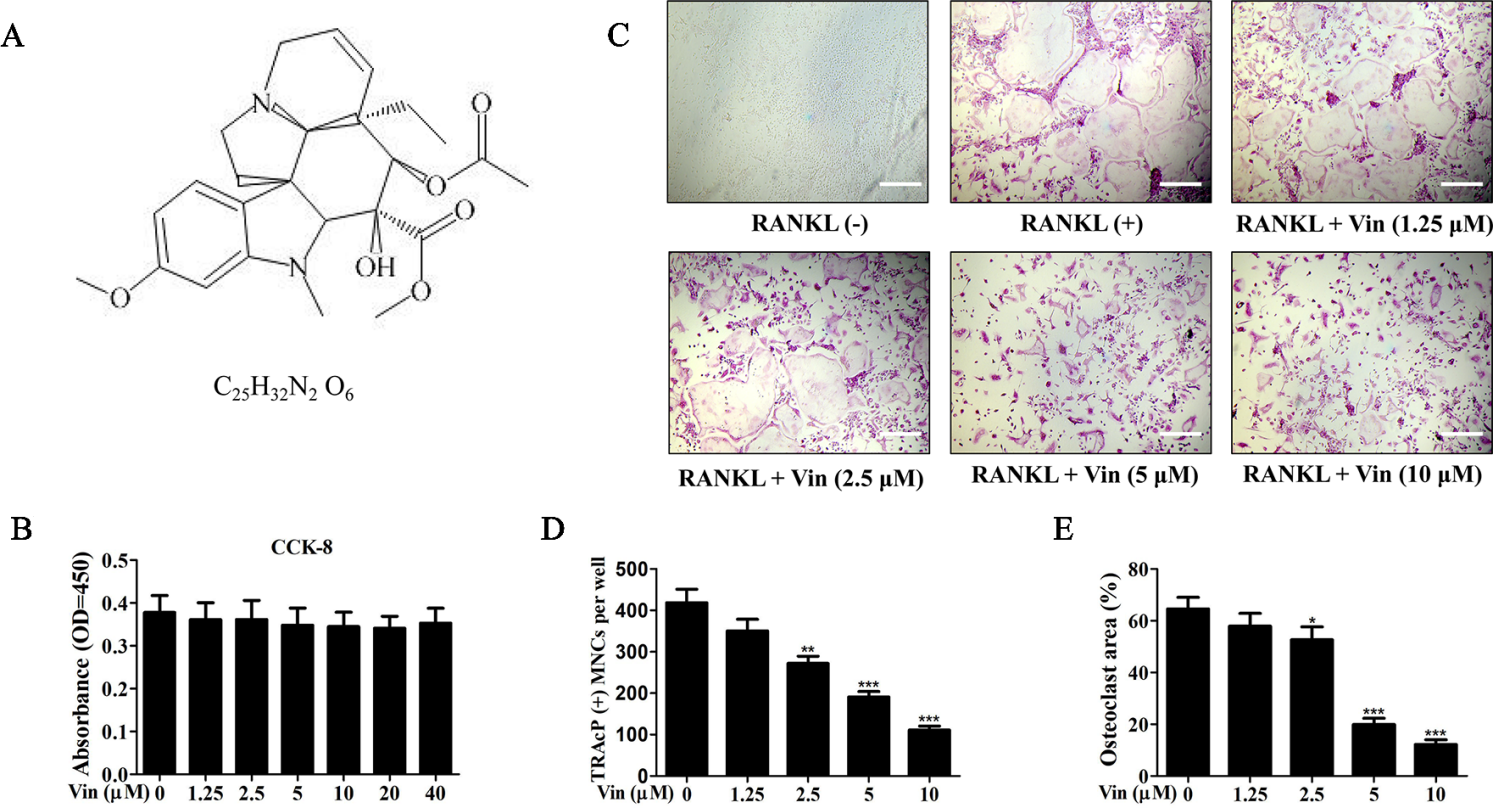
Vin attenuated RANKL‐induced osteoclast formation *in vitro*. **(A)** The chemical structure and formula of Vin. **(B)** Effect of Vin on BMM cell viability. M-CSF-dependent BMMs were treated with indicated concentrations Vin for 48 h and cell viability assessed using the CCK-8 assay (n = 3). **(C)** Representative light micrographs of the dose-dependent effect of Vin on RANKL‐induced osteoclast formation. M-CSF-dependent BMMs stimulated with 100 ng/ml RANKL without or with indicated of Vin for 5 days were fixed and stained for TRAP activity. BMMs without RANKL stimulation and Vin treatment served as untreated mock controls. **(D** and **E)** The number and size (as % area of total well area) of TRAP-positive osteoclasts with 3 or more nuclei were quantified. Data are presented as the mean ± SD; *p < 0.05, **p < 0.01, and ***p < 0.001, relative to RANKL (+) controls. Scale bar, 200 μm.

We first evaluated the cytotoxic effects of Vin (chemical structure shown in **Figure 1A**) on BMM cells. According to the result, Vin had no effect on BMM cell viability even at concentrations of up to 40 μM (**Figure 1B**). We next examined the effect of Vin on osteoclast differentiation. Large “pancake”-shaped multinucleated cells (≥3 nuclei) were considered as mature osteoclasts, which were intensely stained for TRAP activity when stimulated with RANKL, and the number of TRAP-positive osteoclasts decreased in a dose-dependent manner as the concentrations of Vin increased (**Figures 1C–D**). Interestingly, Vin appeared to also inhibit the size (measured in terms of % area) of osteoclasts formed (**Figure 1E**), suggesting that Vin may interfere with fusogenic process of monocytic precursors during osteoclast formation. We also evaluated the effects of Vin on osteoblasts. ALP staining showed no difference between the treatment and control groups. These results indicated that Vin had no effects on osteoblasts (**Supplementary Figure 1**).

**Figure 2:**
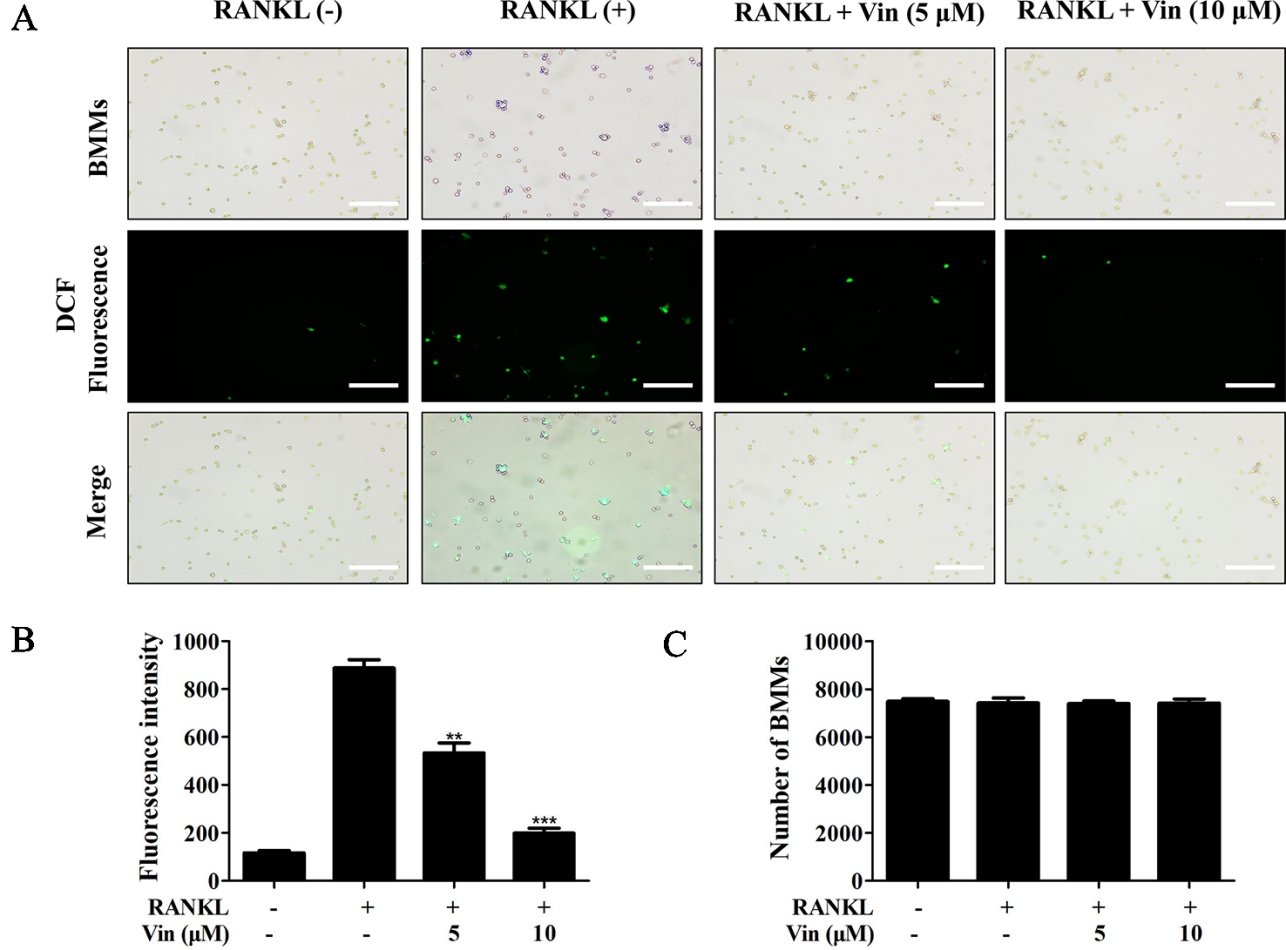
Vin decreased intracellular ROS generation in BMMs. **(A)** Representative fluorescence micrograph of the effect of Vin on RANKL-induced intracellular ROS production. M-CSF-dependent BMMs stimulated with RANKL without or with 5 or 10 μM of Vin for 48 h were incubated with DCFH-DA for 40 min and the oxidative conversion of non-fluorescent DCFH-DA to highly fluorescent DCF was detected under fluorescence microscopy. **(B)** The mean fluorescence intensity of DCF were quantified for each treatment. **(C)** BMMs per well were counted. All data were confirmed in three independent experiments. Data are presented as the mean ± SD; **p < 0.01, and ***p < 0.001, relative to RANKL (+) controls. Scale bar, 200 μm.

Previous reports have shown that ROS plays important roles as secondary messengers in the regulation of osteoclast differentiation (Kim et al., 2017). Here we further showed that Vin treatment dose-dependently inhibited intracellular ROS in response to RANKL stimulation without affecting the number of BMMs (**Figures 2A–C**). In the presence of Vin, the conversion of DCFH-DA to highly fluorescent DCF by ROS was significantly inhibited. Together these data showed that Vin could effectively inhibit RANKL-induced osteoclast differentiation by blocking intracellular ROS production *in vitro*.

### Vin Inhibited Osteoclast Resorptive Function

**Figure 3:**
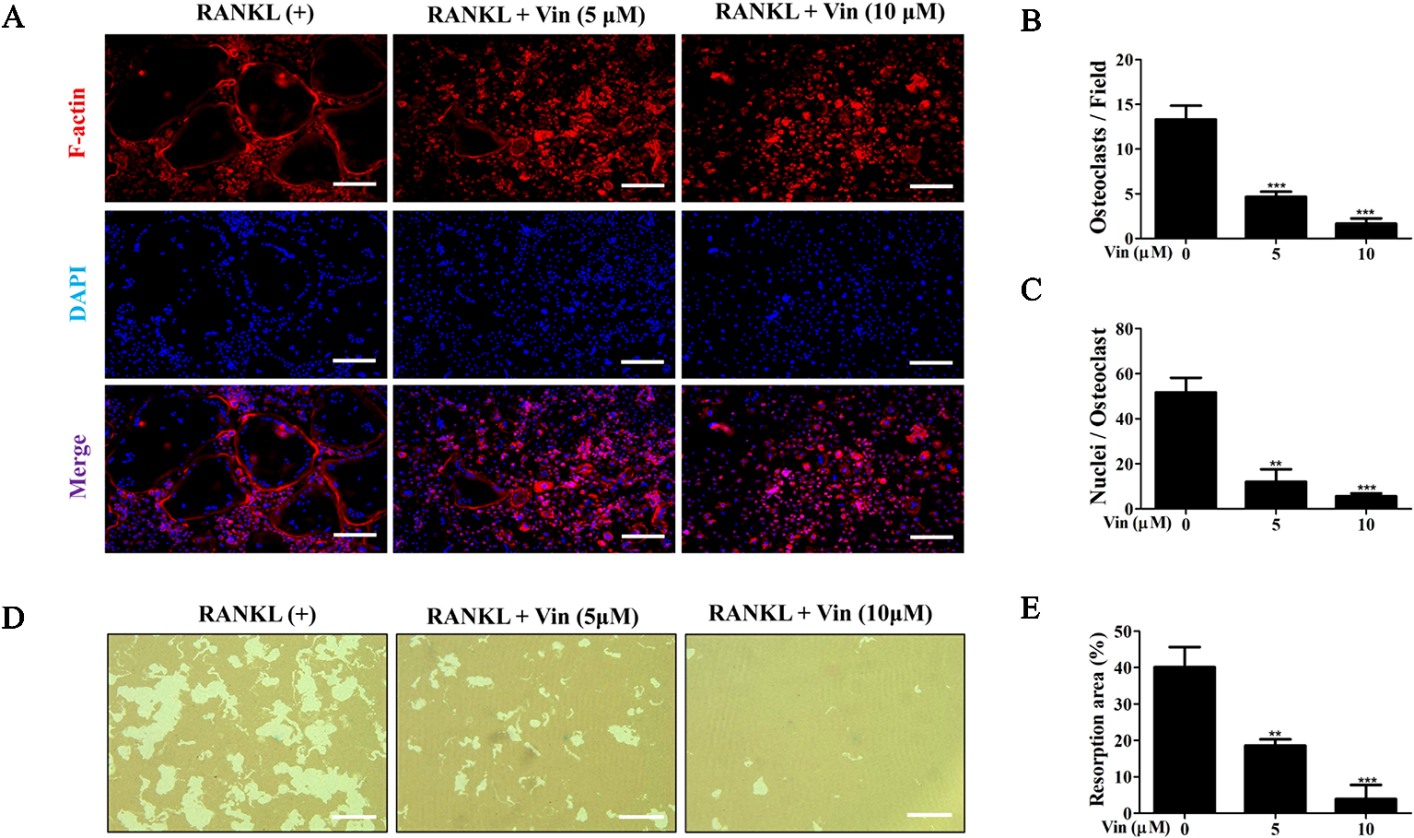
Vin inhibited mature osteoclast bone resorption *in vitro*. **(A)** Representative fluorescence micrographs for the effect of Vin on podosomal F-actin belt formation. BMM-derived osteoclasts stimulated with 100 ng/ml RANKL in the absence or presence of Vin (5 or 10 μM) for 5 days were fixed and actin cytoskeleton stained with Rhodamine-conjugated Phalloidin (red). Nuclei were counterstained with DAPI (blue). **(B)** Quantification of the osteoclasts treated with the indicated concentrations of Vin. **(C)** Quantification of the nuclei number per osteoclast. **(D)** Representative light micrographs of the effect of Vin on the resorptive activity of osteoclasts cultured on hydroxyapatite-coated culture plates. Equal number of pre-osteoclasts stimulated with 100 ng/ml RANKL for 3 days were seeded onto OsteoAssay plates and then treated without or with Vin (5 or 10 μM) for further 48 h. Cells were then removed and resorption pits visualized under light microscopy. **(E)** The percentage of resorption pit area relative to total well area was quantified for each experimental condition; (n = 3). Data are presented as the mean ± SD. **p < 0.01, and ***p < 0.001, relative to RANKL (+) or ‘0' control. Scale bar, 200 μm.

Next, the effects of different concentrations of Vin on osteoclast function were evaluated. Mature osteoclasts were first stained with Rhodamine–phalloidin to assess morphological changes to actin cytoskeletal structure. The F-actin ring is a critical structure of actively resorbing osteoclasts derived from the reorganization of the inactive podosomal actin belt that circumscribes the cell periphery of mature osteoclasts. As shown in **Figure 3**, Vin treatment results in drastic reduction in the size of the podosomal belt consistent with the effect on osteoclast size observed in **Figure 1E** (**Figures 3A, B**). We also observed from the immunofluorescence images that a reduction in multinucleation in cells treated with Vin when compared to RANKL-treated only controls (**Figure 3C**) which further suggests that Vin inhibits precursor cell fusion.

Given that Vin impaired podosomal actin belt formation a prerequisite for osteoclast function, we hypothesized that Vin would also block osteoclast bone resorption. To this end, osteoclast were cultured on hydroxyapatite coated OsteoAssay plates. After 48 h of treatment with or without different concentrations of Vin, resorptive activity of treated osteoclasts were assessed. As shown in **Figures 3D, E**, osteoclasts treated with Vin exhibited significantly reduced ability to effectively resorbed the hydroxyapatite coating. Compared to the control group, where osteoclasts resorbed 40% of total well area, osteoclasts treated with 5 μM of Vin resorbed only 20% of the total well area, whereas 10 μM Vin treatment reduced this down to only 5%. These results indicated that Vin can effectively inhibit mature osteoclast bone resorptive function.

### Vin Blocked RANKL‐Induced Early Activation of MAPK and Subsequent Downstream Induction of NFATc1 During Osteoclast Formation

**Figure 4:**
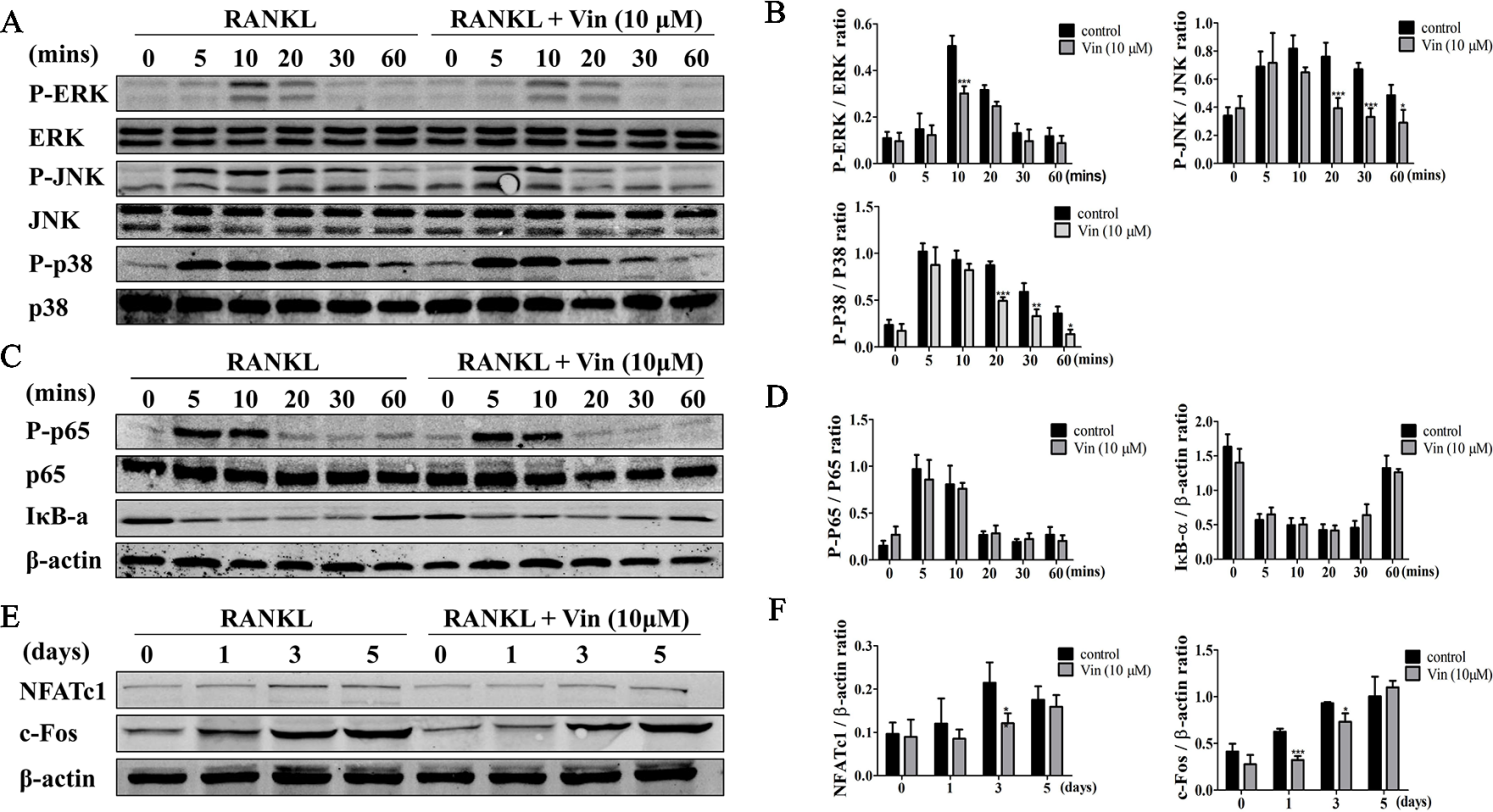
Vin blocked RANKL‐induced activation of MAPK signaling and subsequent NFATc1 induction. **(A)** Representative immunoblots for the effect of Vin on RANKL-induced phosphorylation of ERK, JNK, and p38 MAPKs. Total cellular proteins from BMM pretreated without or with 10 μM Vin for 1 h, and then stimulated with 100 ng/ml RANKL for 0, 5, 10, 20, 30, or 60 min were subjected to western blot analyses using specific antibodies against total and phosphorylated forms of ERK, p38, and JNK. **(B)** Relative changes in the phosphorylation status of ERK, JNK, and p38 relative to their respective total protein counterpart were quantified by densitometry; (n = 3). **(C)** Representative immunoblots for the effect of Vin on RANKL-induced degradation of IκBα and phosphorylation of NF-κB p65. Total cellular proteins from experimental conditions above were analyzed by western blot using specific antibodies against IκBα, and total and phosphorylated forms of p65. **(D)** Relative changes in the phosphorylation status of p65 relative to total p65, and total protein levels of IκBα relative to β-actin were quantified by densitometry; (n = 3). **(E)** Representative immunoblots for the effect of Vin on RANKL-induced expression of c-Fos and NFATc1. Total cellular proteins from BMM stimulated with RANKL without or with 10 μM Vin for 0, 1, 3, or 5 days were subjected to western blot analyses using specific antibodies against c-Fos and NFATc1. **(F)** Relative expression of c-Fos and NFATc1 relative to β-actin were quantified by densitometry; (n = 3). β-actin was used as internal loading control for all experimental conditions. Data are presented as the mean ± SD; *p < 0.05, **p < 0.01, and ***p < 0.001.

RANKL binds to the receptor RANK activates a cascade of signaling events *via* the adaptor protein TRAF6 and second messenger systems such as ROS (Boyle et al., 2003; Callaway and Jiang, 2015). Of the signaling pathways activated, NF-κB and MAPK are important in early phase of osteoclast differentiation process (Takayanagi, 2005). Hence we analyzed the impact of Vin treatment on the early activation of these two signaling cascades. The results revealed that the activation phosphorylation of ERK, JNK and p38 occur within minutes of RANKL stimulation–within 10 min and lasting 20 min for ERK, and within 5 min and lasting 60 min for both JNK and p38 (**Figures 4A, B**). On the other hand, Vin treatment significantly blocked the phosphorylation of all three MAPK members (ERK, JNK, and p38) (**Figures 4A, B**). The activation of NF-κB signaling involves the degradation of inhibitory IκBα which functions to retain p65/p50 NF-κB subunits in the cytoplasm. The degradation of IκBa occurs within minutes of RANKL stimulation and lasting for over 30 min (**Figures 4C, D**). During this time p65 is activated by phosphorylation enabling it to translocate from the cytoplasm to the nucleus to initiate transcription of target genes. Unlike the inhibitory effect on MAPK signaling, Vin treatment did not affect IκBα degradation and p65 activation phosphorylation. In conclusion, Vin had little effect on the NF-κB pathway but inhibited the RANKL-induced MAPK signaling pathway in osteoclasts.

### Vin Repressed the Induction of NFATc1 and Expression of NFATc1 Target Genes

Next, protein extracts from BMM-derived osteoclasts treated without or with Vin for 1, 3, or 5 days were examined for the expression of c-Fos and NFATc1. As shown in **Figure 4E**, the induction of c-Fos at day 1 following RANKL stimulation precedes that of NFATc1 induction which occurs around day 3 and lasting throughout osteoclastogenesis. This expression pattern of NFATc1 coincides with the period where monocytic precursor cells are undergoing cellular fusion and maturation into multinucleated osteoclasts. In line with the blockade in MAPK signaling, both c-Fos and NFATc1 were significantly repressed following Vin treatment (**Figures 4E, F**).

**Figure 5:**
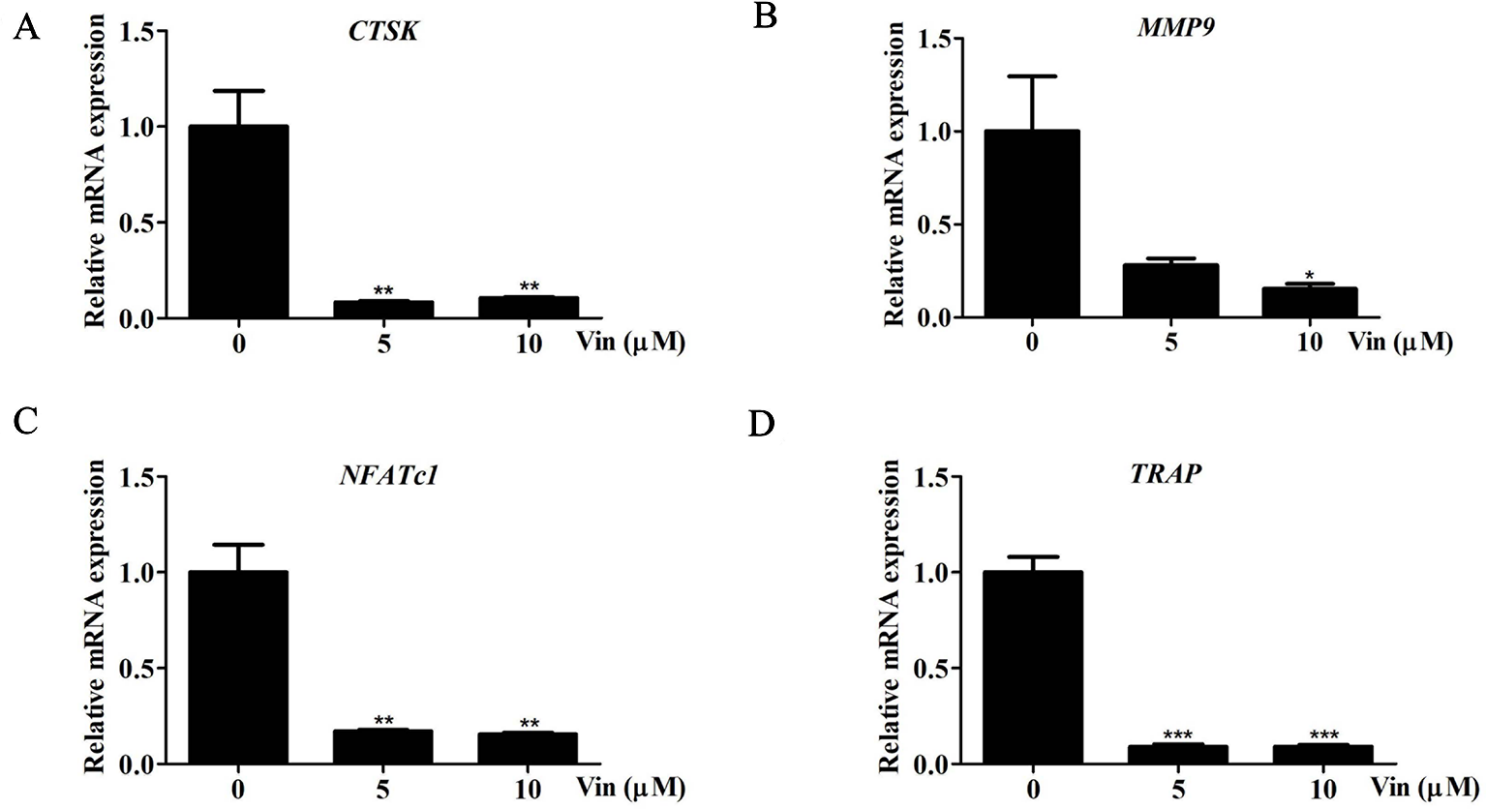
Vin reduced the expression of osteoclast marker genes. **(A–D) **Real-time qPCR was performed on RNA extracted from cells stimulated with 100 ng/ml RANKL without or with indicated concentrations of Vin for 5 days. The expression levels of CTSK, MMP9, NFATc1, and TRAP were normalized to GAPDH and then compared to RANKL (+) or “0” control to obtain relative fold change; (n = 3). Data are presented as the mean ± SD; *p < 0.05, **p < 0.01, and ***p < 0.001.

Consistent with repression of NFATc1 induction, the expression of NFATc1, *CTSK*, *MMP9,* and *TRAP* were similarly downregulated following Vin treatment (**Figures 5A–D**) further strengthening the evidence that Vin is an effective inhibitor of osteoclast formation and function. Collectively our biochemical analysis provides a better understanding on the mechanism by which Vin inhibited osteoclast formation and function, i.e. *via* the blockade of MAPK activation and repressing NFATc1 induction.

### Vin Lessened the Deleterious Effect of OVX-Induced Bone Loss in Mice

**Figure 6:**
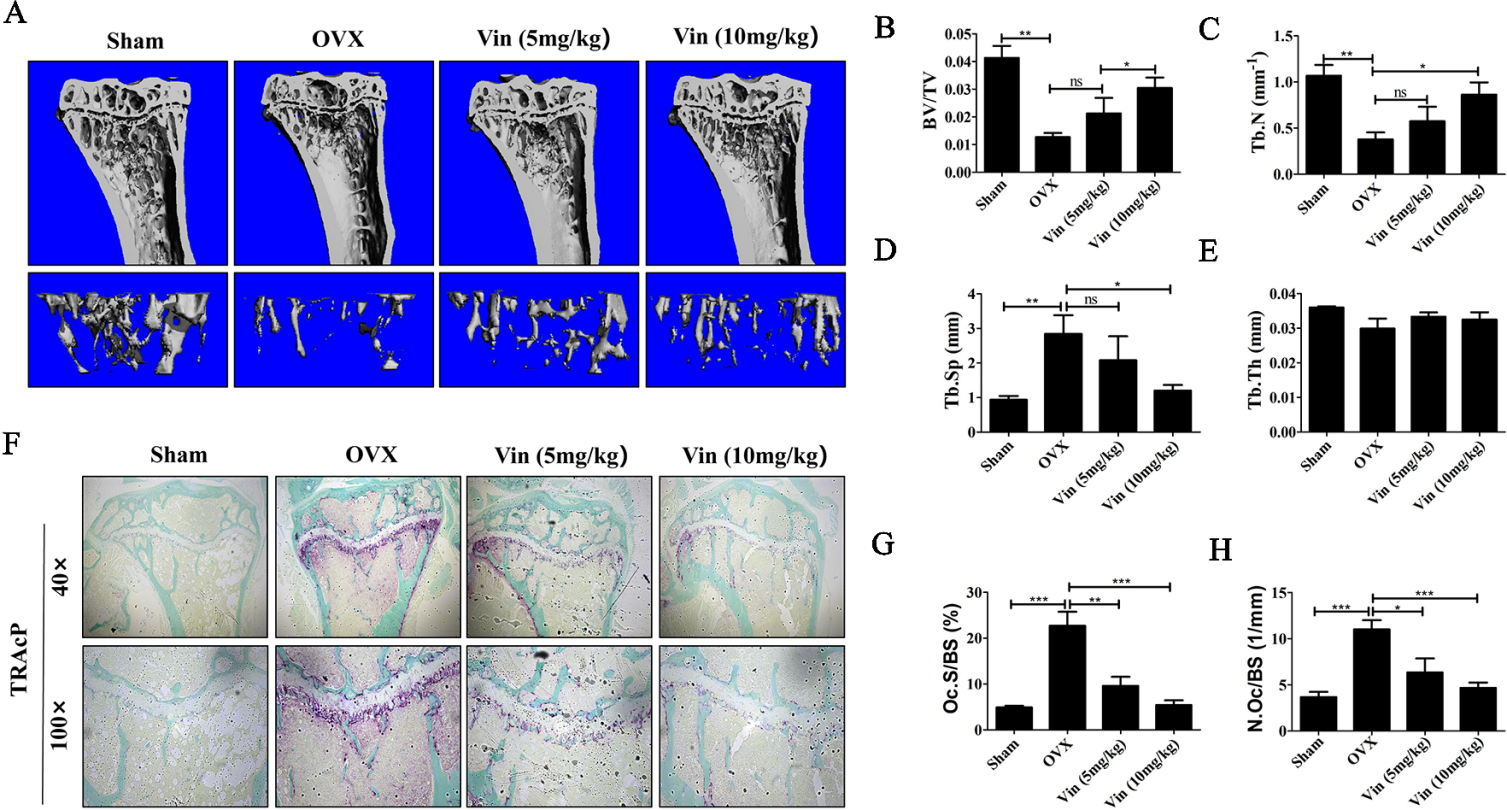
Vin lessened the deleterious effect of OVX-induced bone loss in mice. **(A)** Representative 3D reconstructions of micro‐CT scans of the tibia from Sham, OVX, OVX + low-dose Vin (5 mg/kg body weight), and OVX + high-dose Vin (10 mg/kg body weight). **(B–E)** Quantitative analyses of morphometric bone parameters of bone volume to tissue volume (BV/TV), trabecular number (Tb. N), trabecular separation (Tb. Sp) and trabecular thickness (Tb. Th) of each experimental condition. **(F)** Representative images of decalcified tibial bone tissue from mice in each experimental groups stained for TRAP activity. **(G–H)** Quantitative analyses of N.Oc/BS and Oc.S/B. Data are expressed as means ± SD. ns: no significance, *p < 0.05, **p < 0.01, and ***p < 0.001 relative to respective control group.

With promising *in vitro* results, we next established an *in vivo* OVX model to further validate the effect of Vin on osteoclast activity. Estrogen deficiency-mediated bone loss underlies postmenopausal osteoporosis in humans. Here in our murine model, 6-weeks after bilateral OVX, compared with Sham-operated mice, micro-CT 3D reconstructions of tibial bones showed marked reduction in bone volume and significant deterioration in trabecular bone architecture in experimental OVX mice (**Figure 6A**). On the other hand, Vin administration in particular high-dose (10 mg/kg body weight) treatment, considerably lessened the deleterious effect of OVX, protecting mice against bone loss and trabecular bone changes. Quantitative morphometric analysis of bone volume (BV/TV), and trabecular bone parameters (Tb. N, and Tb. Sp) further confirmed our micro-CT findings (**Figures 6B–E**). However, no significant effect was measured for Tb. Th in both OVX and OVX + Vin treated mice. By histological assessment of osteoclast number and activity by TRAP, we were able to see that OVX radically induced osteoclast formation and bone resorption when compared to Sham mice and this elevation in osteoclast number and activity was dose-dependently reduced following Vin administration (**Figures 6F–H**). Thus, taken together, our *in vivo* data provided additional evidence to support the potential use of Vin in the effective treatment or management of osteoclast-mediated osteolytic bone diseases such as postmenopausal osteoporosis.

## Discussion

Many osteolytic bone disease such as osteoporosis is often caused by elevated osteoclast formation and/or overactivation of bone resorptive activity (Li et al., 2019). This leads to substantial decrease in bone volume, reduced bone density and harmful deterioration of bone microarchitecture that consequently results in increased bone fragility, which is prone to fracture (Johnell and Kanis, 2006; Bartelt et al., 2018). Therefore, osteoclasts remain the primary cellular target for the identification and development of therapeutic agents that are effective in the treatment of osteoporosis. Currently, the therapeutic agents used clinically to treat osteoporosis are selective estrogen receptor modulators (SERMs), Denosumab (anti-RANKL antibody), bisphosphonates, parathyroid hormone, and health supplements including calcium and vitamin D (Cranney et al., 2002; Zur et al., 2018). However, side effects such as nephrotoxicity, osteonecrosis, endometrial cancer risk, and jaw bone necrosis limit the use of these agents (Khan et al., 2017). In recent years, interest in naturally-derived chemical agents and compounds has surged due to their wide range of biological and pharmacological effects, some of which possesses anti-osteoclastogenic and anti-resorptive properties (Chen et al., 2019). Our current study demonstrated that Vin, an indole alkaloid extracted from the medicinal plant *Catharanthus roseus*, can prevent OVX-induced bone loss *in vivo* by inhibiting MAPK signaling pathway and intracellular ROS production which consequently represses the induction NFATc1 during osteoclast differentiation.

Efficient osteoclast formation requires timely and unhindered activation of several key signaling pathways throughout the differentiation process (Theill et al., 2002). Of these, the MAPK and NF-κB signaling cascades are activated during the very early phase of RANKL stimulation. In resting unstimulated cells, NF-κB p65/p50 subunit dimers are retained in the cytoplasm (Hansen et al., 1994). Binding of RANKL to receptor RANK rapidly induces IκBα phosphorylation by IκB kinases (IKKs) leading to its subsequent degradation *via* the proteasomal degradative pathway (Nemeth et al., 2004; Mathes et al., 2008). This allows NF-κB p65 subunit to be activated by phosphorylation and transferred from the cytoplasm to the nucleus to transcriptionally activate the target gene (Xiu et al., 2014). Despite its importance in osteoclast formation, Vin treatment did not inhibit RANKL-induced IκBα degradation or p65 phosphorylation and therefore did not affect the activation of NF-κB signaling.

Concurrent with NF-κB activation, the MAPK signaling cascade consisting of three signaling members ERK, JNK and p38, are also activated by RANKL (Koh et al., 2006). Although all three MAPK members are required for effective osteoclast formation, JNK and ERK have also been shown to modulate osteoclast survival, whilst ERK also plays an important role in maintaining the polarity of osteoclasts during bone resorption (Li et al., 2002). Dephosphorylation of the threonine and/or tyrosine residues by various phosphatases such as dual-specificity MAPK phosphatases (MKPs), serine/threonine phosphatases and tyrosine phosphatases inactivate the activated MAPK (Bardwell et al., 2003). Interestingly, elevated ROS has been shown to inhibit MKPs function *via* oxidation and in recent years, RANKL-induced intracellular ROS production have been proved to be important second messengers in the regulation of efficient osteoclast formation (Kamata et al., 2005; Yip et al., 2005). We found that Vin treatment resulted in reduced phosphorylation of all three MAPK members and attenuated intracellular ROS production suggesting elevated actions of MKPs could be in part responsible for the inhibition of MAPK activation during RANKL-induced osteoclast formation. However, the exact MKP responsible for this effect will require further exploration.

The expression of c-Fos precedes NFATc1 and plays a role in the induction of NFATc1 *via* the activator protein-1 (AP-1) transcriptional complex (c-Fos/c-Jun dimers) (Wagner, 2010). Mice deficient in c-Fos or mice over-expressing a dominant negative c-Jun exhibits severe osteopetrosis on account of lack of osteoclast formation demonstrating the importance of the AP-1 transcriptional complex in osteoclast formation (Arai et al., 2012). In our study we showed that cells that were treated with Vin had repressed expression of c-Fos during the early (day 1) and intermediate stages (day 3) of osteoclast differentiation which is consistent with impaired MAPK activation.

The robust expression of NFATc1 during the intermediate and terminal stages of osteoclast differentiation is necessary for NFATc1-dependent transcriptional activation of target genes involved in osteoclast fusion and bone resorption (Kim et al., 2008). We showed in our biochemical and gene expression analysis that the induction of NFATc1 expression was delayed during osteoclast differentiation which consequently resulted in the downregulation of various NFAT-responsive genes involved in osteoclast fusion including CTSK, MMP9, and TRAP. The downregulation of the latter genes was in line with our cellular experiments showing the inhibitory effects of Vin on mature osteoclast bone resorption. We observed decreased formation of podosomal F-actin belt in mature osteoclasts which correlated with impaired hydroxyapatite resorption activity following Vin treatment. Collectively, based on our cellular and biochemical analyses we hypothesize that the underlying mechanism *via* which Vin depresses osteoclast formation and function is in part due to the suppression of MAPK activation and intracellular ROS production which consequently represses the induction of c-Fos and NFATc1. Nevertheless, a more in-depth analysis of the target and the potential mechanisms of Vin in the osteoclasts is required.

Osteoblasts were the main cells involved in bone formation and sever as an irreplaceable role in maintaining bone mass. Therefore, we investigated the effect of Vin on osteoblasts. Our results indicated that Vin exerts no effect on the differentiation of osteoblasts.

Considering the effect of Vin *in vitro*, we further examined the potential benefits of Vin treatment in bone loss induced by OVX mice model *in vivo*. This animal model is a well-established model for the investigation of effects of biological and chemical compounds estrogen deficiency-induced bone loss and closely mimics the characteristics and bone changes associated with postmenopausal osteoporosis (Komori, 2015). Micro CT and morphometric analysis showed that the administration of Vin particularly at high-dose (10 mg/kg of body weight) protected mice against the deleterious effect of OVX by enhancing bone volume and restoring trabecular bone microarchitecture. Histological assessments confirmed our *in vitro* data, showing that Vin treatment reduced the number of TRAP-positive osteoclasts present in bone suggesting that the protective effect of Vin was in part due suppression of osteoclast-mediated bone destruction. However, given that Vin has been previously reported to possess anti-inflammatory and antioxidant effects, we cannot rule out the possibility that these properties could have contributed to the biological effects of Vin on OVX-induced bone loss. Nonetheless, our study demonstrates for the first time that Vin possesses anti-osteoclastogenic and anti-resorptive properties which can be exploited to our benefit for the management or treatment of osteoclast-mediated osteolytic diseases such as osteoporosis.

## Data Availability Statement

The data that support the findings of this study are available from the corresponding author upon reasonable request.

## Ethics Statement

All experiments were approved by the Ethics Committee of Guangxi Medical University. All animal experiments and surgical procedures were performed according to the guidelines of the Animal Care Committee of Guangxi Medical University.

## Author Contributions

YZ and JL conducted research and drafted the manuscript. KT, ZC, ZW, XY, YS, XL, FS, and JZ provided valuable opinions, evaluation, and assistance in the process of drafting and revision of the manuscript. JX, QL, and BZ supervised the study and revised the manuscript.

## Funding

This study was supported by Guangxi Science and Technology project (Guikehe1599005-2-12), Guangxi Science and Technology Department funded project (AD17195086), Guangxi Natural Science Foundation, (2018GXNSFAA294053, 2018GXNSFAA050092), and the Guangxi Collaborative Innovation Center for Biomedicine talent cultivation (GCICB-TC-2017001, GCICB-TC-2017002). This study was also funded by the Australian Health and Medical Research Council (NHMRC No: APP1107828). Funding statement is not included in the manuscript.

## Conflict of Interest

The authors declare that the research was conducted in the absence of any commercial or financial relationships that could be construed as a potential conflict of interest.
