# Supplementary figures and images for: Vindoline Inhibits RANKL-Induced Osteoclastogenesis and Prevents Ovariectomy-Induced Bone Loss in Mice
Source: Front Pharmacol. 2020 Jan 22;10:1587. doi: 10.3389/fphar.2019.01587 (PMC6987431; doi:10.3389/fphar.2019.01587)

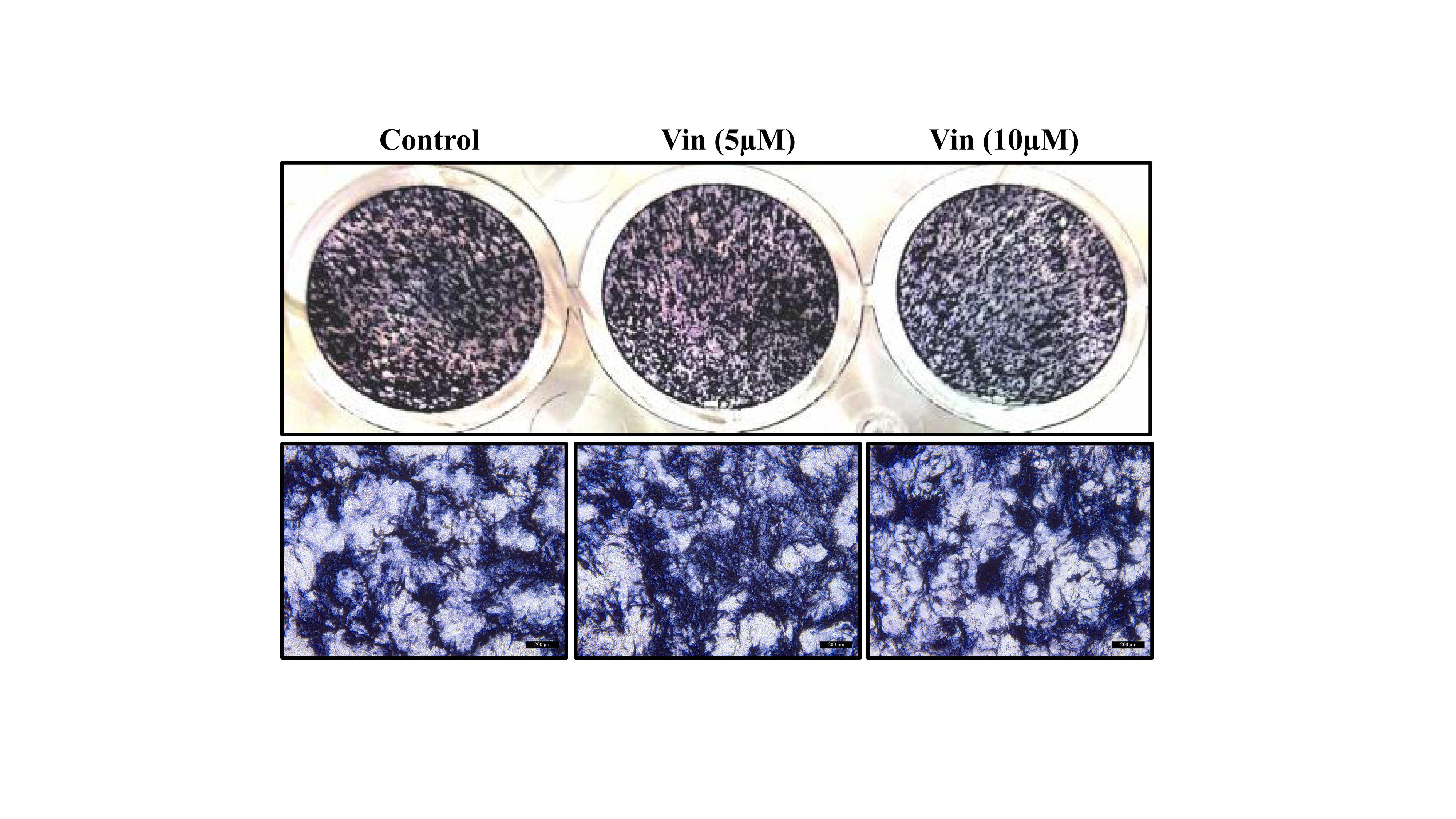

Supplement: Supplementary file 1 [file Image_1.tif]
