# Supplementary material for: Vindoline Inhibits RANKL-Induced Osteoclastogenesis and Prevents Ovariectomy-Induced Bone Loss in Mice
Source: Front Pharmacol. 2020 Jan 22;10:1587. doi: 10.3389/fphar.2019.01587 (PMC6987431; doi:10.3389/fphar.2019.01587)
Supplement: Supplementary file 2 [file Table_1.doc]

**TABLE1** Primer sequences for q‐PCR

| **Genes                                        Primer sequences** |
| --- |
| TRAcP/Acp5                  Forward  5ʹACGGCTACTTGCGGTTTCA-3ʹ                                         Reverse   5ʹ-TCCTTGGGAGGCTGGTCTT-3ʹ |
| MMP9                            Forward  5ʹ-GAAGGCAAACCCTGTGTGTT-3ʹ                                         Reverse   5ʹ-AGAGTACTGCTTGCCCAGGA-3ʹ |
| CTSK                             Forward  5ʹ-AGGCGGCTATATGACCACTG-3ʹ                                         Reverse   5ʹ-TCTTCAGGGCTTTCTCGTTC-3ʹ |
| NFATc1                           Forward  5ʹ-GGTGCTGTCTGGCCATAACT-3ʹ                                        Reverse   5ʹ-GAAACGCTGGTACTGGCTTC-3ʹ |
| GAPDH                        Forward  5ʹ-AACTTTGGCATTGTGGAAGG-3ʹ                                        Reverse  5ʹ-ACACATTGGGGGTAGGAACA-3ʹ |
